# Tumor budding is an independent prognostic marker in early stage oral squamous cell carcinoma: With special reference to the mode of invasion and worst pattern of invasion

**DOI:** 10.1371/journal.pone.0195451

**Published:** 2018-04-19

**Authors:** Shota Shimizu, Akihiro Miyazaki, Tomoko Sonoda, Kazushige Koike, Kazuhiro Ogi, Jun-ichi Kobayashi, Takeshi Kaneko, Tomohiro Igarashi, Megumi Ueda, Hironari Dehari, Akira Miyakawa, Tadashi Hasegawa, Hiroyoshi Hiratsuka

**Affiliations:** 1 Department of Oral Surgery, Sapporo Medical University School of Medicine, Sapporo, Japan; 2 Department of Public Health, Sapporo Medical University School of Medicine, Sapporo, Japan; 3 Department of Surgical Pathology, Sapporo Medical University School of Medicine, Sapporo, Japan; Charles P. Darby Children’s Research Institute, UNITED STATES

## Abstract

Pathologically proven regional lymph node metastasis affects the prognosis in early stage oral cancer. Therefore we investigated invasive tumor patterns predicting nodal involvement and survival in patients with clinically node-negative T1 and T2 oral squamous cell carcinoma (cT1,2N0M0 OSCC). Ninety-one cases of cT1,2N0M0 OSCC treated with transoral resection of the primary tumor were assessed based on 3 types of invasive tumor patterns on histopathologic and pancytokeratin-stained immunohistological sections: the mode of invasion, worst pattern of invasion (WPOI), and tumor budding. The correlations among invasive tumor patterns, regional metastasis, and disease-free survival were analyzed. Of the 91 cases, 22 (24%) had pathologically proven regional metastasis. The mode of invasion (p<0.01) and tumor budding (p<0.01) were associated with regional metastasis as well as lymphovascular invasion (p = 0.04) in univariate analysis. In logistic regression analysis, however, tumor budding was the only independent predictor of regional metastasis (hazard ratio (HR) = 3.05, 95% confidence interval (CI) = 0.29–5.30, p<0.01). All three invasive patterns, the mode of invasion, WPOI, and tumor budding, were found to be significant predictors of 5-year disease-free survival (p<0.01, p = 0.03, and p<0.01, respectively) as well as lymphovascular invasion (p = 0.02) and perineural invasion (p = 0.02). A final model for Cox multivariate analysis identified the prognostic advantage of the intensity of tumor budding (HR = 2.19, 95% CI = 1.51–3.18, p<0.01) compared with the mode of invasion and WPOI in disease-free survival. Our results indicate that the intensity of tumor budding may be a novel diagnostic biomarker, as well as a therapeutic tool, for regional metastasis in patients with cT1,2N0M0 OSCC. If the pancytokeratin-based immunohistochemical features of more than five buds, and a grade 4C or 4D mode of invasion are identified, careful wait-and-see follow-up in a short period with the use of imaging modalities is desirable. If there are more than ten buds, a grade 4D mode of invasion, or WPOI-5 in the same section, wide resection of the primary tumor with elective neck dissection should be recommended.

## Introduction

Although oral squamous cell carcinomas (OSCC) constitute a broad range of tumors with diverse etiologies, the estimated age-standardized rate is relatively large, at 2.7 per 100,000 (3.7 for men and 1.8 for women) in 2012 [1]. Transoral surgical excision of the primary tumor represents the mainstay of treatment for patients with early stage clinically node-negative OSCC. However, these early stage diseases have a high risk of regional metastasis, which can exceed 30% [2, 3]. Regional lymph node status is the most important predictor of survival for OSCC patients. Despite recent advances in the clinical evaluation of regional metastasis in early stage OSCC, including imaging techniques such as computed tomography (CT) scanning, magnetic resonance imaging (MRI), ultrasonography (US), positron-emission tomography (PET)-CT, and serum tumor markers, it cannot always be determined. Despite this high risk of occult disease, the issue of whether to recommend elective neck dissection for patients with clinically node-negative disease represents an area of historic controversy. A recent prospective randomized controlled trial revealed that elective neck dissection resulted in higher rates of overall survival and disease-free survival than did therapeutic neck dissection for clinically node-negative T1 and T2 (cT1,2N0M0) OSCC [3]. However, it produces unnecessary morbidity in OSCC patients without metastases. Indeed, the trial results mean that 8 patients would need to be treated with elective neck dissection to prevent one death, and four patients would need to be treated to prevent one nodal relapse (the development of nodal disease after the excision of the primary tumor in patients without elective neck dissection) as pointed out by the authors.

Many studies of potential molecular and morphologic predictors for regional metastasis in clinically node-negative OSCC have been published. However, none of them have as yet achieved a general clinicial consensus [4]. These facts point to the immediate need for new diagnostic-prognostic strategies and additional practical parameters that will improve the clinical decision-making and management of patients with cT1,2N0M0 OSCC. Recently, evidence has accumulated that high tumor budding at the tumor invasive front of OSCC is associated with regional metastasis and poorer survival [5]. Indeed, it has long been considered that cancer cells located in the tumor–host interface of OSCC tissues are more aggressive in terms of metastatic potential [6, 7]. Based on an experimental autochthonous tumor rejection model in rats, it was already pointed out that if the tumor-bearing hosts had specific immune resistance to their own cancer, the site of the cancer invasion and spread would be a battlefield [8]. This battlefield is currently termed the deep invasive front, advancing tumor edge, or advancing front of the tumor [9]. The invasive front of OSCC has been an area of histopathologic research interest [10–13].

To identify tumor patterns at the invasive tumor front predicting regional metastasis in cT1,2N0M0 OSCC, we investigated three histopathologic and immunohistopathologic invasive tumor patterns that are observed easily in routine hematoxylin and eosin (H&E)-stained sections and/or cytokeratin-immunostained sections: the mode of invasion, worst pattern of invasion (WPOI), and tumor budding. The mode of invasion classification proposed by Yamamoto et al. [11] in which grade 4 of the criteria of Jakobsson et al. [10] was subclassified into grade 4C (diffuse invasion of the cord-like type) and grade 4D (diffuse invasion of diffuse type), is a potentially powerful parameter predicting regional metastasis in patients with clinically node-negative OSCC [13, 14]. The draft proposal for General Rules for Clinical and Pathological Studies on Oral Cancer of the Japan Society for Oral Tumors recommends that the mode of invasion should be recorded as one of the valuable pathologic findings [15]. The WPOI was proposed by Brandwein et al. in 2005 [16]. WPOI-4 tumors are defined as having small tumor islands (≤15 cells per island) that are discontinuous, or convincingly separated from the main tumor mass. WPOI-5 tumors are recognized by a dispersed, discontinuous growth pattern, and the degree of tumor dispersion exceeds that seen for WPOI-4 tumors with a defined cutoff of 1 mm. The new version of the AJCC staging system indicates that the WPOI-5 assessed at the advancing tumor edge is an important prognosticator in oral cancer [17]. On the other hand, tumor budding is defined as a single cell or small clusters of tumor cells at the invasive tumor front. Tumor budding has previously been demonstrated to be a valuable prognostic marker for colorectal cancer and is presented in the colon and rectum section of the UICC Cancer Staging Manual, 8^th^ edition, as a prognostic tumor parameter [18, 19]. However, it is sometimes difficult to detect budding foci by conventional pathologic examination of H&E-stained sections alone because tumor buds can be very small and resemble the surrounding stromal cells [9, 20]. Furthermore, the distribution and frequency of budding foci differs even among tumors with the same budding grade. Accordingly, we attempted to detect the budding cells using immunohistochemical pancytokeratin staining. In addition, we also investigated the contribution of the invasive tumor pattern to a high risk of regional metastasis and survival in patients with cT1,2N0M0 OSCC in the present study.

## Material and methods

### Patients

Diagnostic tissue blocks for patients with cT1,2N0M0 OSCC who had undergone transoral tumor ablation surgery with or without elective neck dissection between January 2004 and December 2013 at the Sapporo Medical University Hospital were collected from the hospital’s archives and used in this study. None of the patients received any forms of neoadjuvant therapy prior to surgery. The use of patient samples and the data inquiry were approved by the Ethics Committee of Sapporo Medical University (No. 292–1044). All study participants provided informed consent. The tumor extent and the histopathological grading were classified according to the seventh edition of the AJCC/UICC TNM Classification [21, 22].

### Immunohistopathologic analysis

Immunohistochemistry was employed to detect multi-cytokeratins and identify the tumor cells. Briefly, 4 um serial sections from paraffin-embedded samples deparaffinized in xylene were soaked in 10mM citrate buffer (pH 8.0) and placed in an autoclave at 121°C for 10 minutes for antigen retrieval. Endogenous peroxidase was blocked by incubation with 0.3% hydrogen peroxide in methanol for 30 minutes. The sections were then incubated with a primary monoclonal antibody targeting pancytokeratin (1:200, clone AE1/AE3, abcam) at 4°C overnight. Secondary antibodies were applied as indicated for the EnVision system (EnVision^+^; DAKO, Glostrup, Denmark). Staining was visualized with diaminobenzidine tetrachloride. The sections were counterstained with hematoxylin, dehydrated, cleared, and mounted. Next, serial sections from the paraffin-embedded samples were stained with H&E and provided for assessment of the mode of invasion and WPOI. The presence and absence of lymphovascular invasion and perineural invasion were tabulated according to information from routine histopathological reports.

### Evaluation of invasive tumor patterns

**Fig 1:**
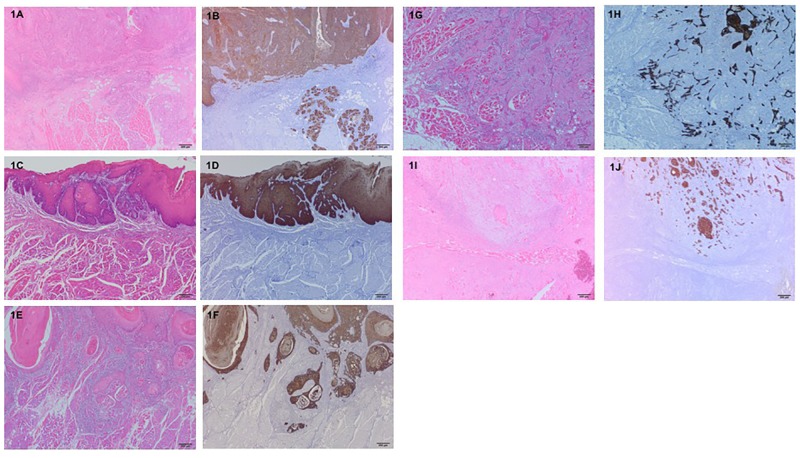
Representative photographs of classification of mode of invasion (H&E staining, immunohistochemical pancytokeratin staining, x40 magnification). (1A,1B) grade 1, (1C,1D) grade 2, (1E,1F) grade 3, (1G,1H) grade 4C, and (1I,1J) grade 4D.

**Fig 2:**
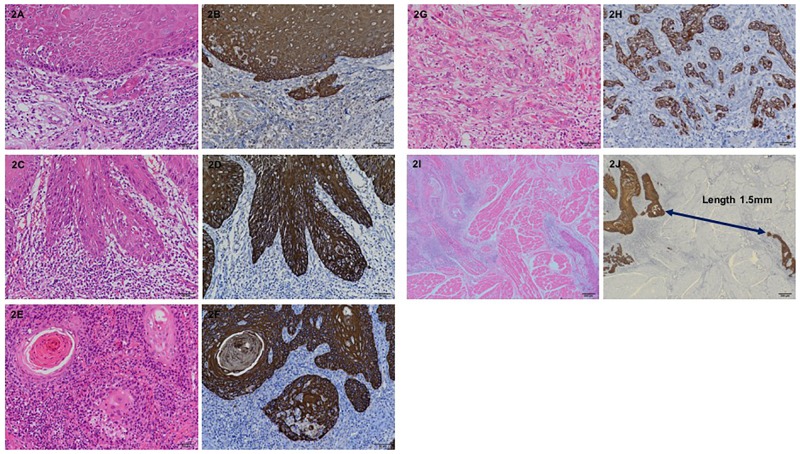
Representative photographs of classification of worst pattern of invasion (H&E staining, immunohistochemical pancytokeratin staining, x200 magnification). (2A,2B) Worst pattern of invasion (WPOI)-1, (2C,2D) WPOI-2, (2E,2F) WPOI-3, (2G,2H) WPOI-4, and (2I,2J, x40 magnification) WPOI-5.

**Fig 3:**
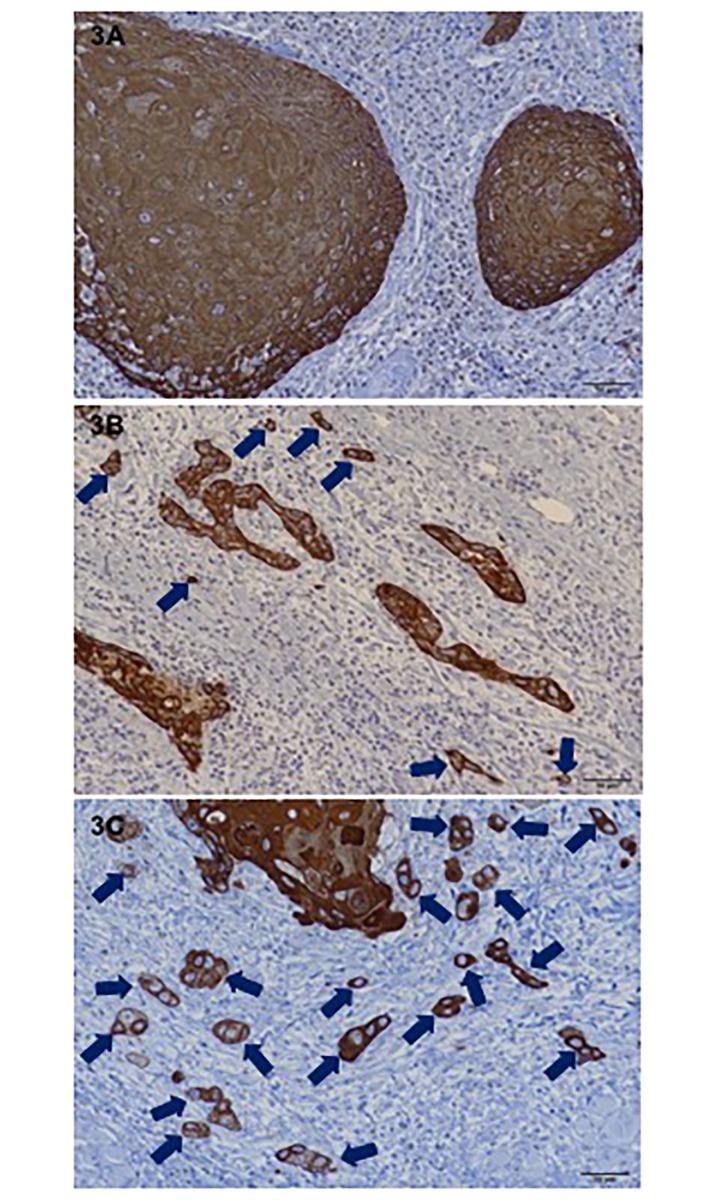
Representative photographs of intensity of tumor budding (immunohistochemical pancytokeratin staining, x200 magnification). (3A) Low intensity of tumor budding (<5 buds), (3B) intermediate intensity of tumor budding (≥5~<10 buds), and (3C) high intensity of tumor budding (≥10 buds).

The mode of invasion and WPOI were assessed on H&E stained sections, and the localization and distribution of the pan-cytokeratin-positive cancer cells were confirmed on immunohistochemically stained sections. The histopathologic evaluation of the mode of invasion at the invasive front of the tumor was done according to Yamamoto et al. [11]. In this assessment, the mode of invasion is graded 1 to 4D. Grade 1 tumors have well-defined borderlines. Grade 2 tumors have cords and less marked borderlines. Grade 3 tumors have groups of cells and no distinct borderlines. Grade 4C tumors have diffuse invasion like cord-like type invasion. In grade 4C, tumor cells invade deeply as a cord-shaped microtumor nest. In grade 4D tumors, a few or single tumor cells invade the deeper portion diffusely (Fig 1). WPOI was assessed according to previous descriptions (Fig 2) [16, 23]. Tumor budding was defined as the presence of isolated single tumor cells or a small group of fewer than five cells ahead of the deep invasive tumor parts (Fig 3). Immunostained tumor specimens were initially scanned with a x4 objective lens (and x10 ocular one) to select the areas with the highest density of budding. Tumor budding in the selected areas was then counted using a x20 objective lens, and the highest count per slide was used as the number of buds. The intensity of tumor budding was arbitrarily categorized into low intensity (<5 buds/field), intermediate intensity (≥5~<10 buds/field) and high intensity (≥10 buds/field) according to the ITBCC 2016 recommendations [24]. The low intensity group included sections with no tumor bud detectable. In evaluating each microscopic feature, special attention was paid to peripheral areas of the most invasive carcinoma mass. If different grades of each criteria were observed in the same specimen, the higher grade was taken as representative. These invasive tumor patterns were assessed on a personal computer with DP2-BSW software for an Olympus Microscope Digital Camera (Olympus Co., Tokyo) by three of the authors (SS, AM, and KK) together at the same time. The labels bearing the patients’ names were covered.

### Statistical analysis

The chi-square test and Fisher’s exact test were used to estimate correlations among the mode of invasion, WPOI, the tumor budding, various histological parameters, and regional metastasis. Disease-free survival defined as the time from the date of surgery to the date of evidence of tumor relapse at any site, including primary and secondary ones, death from any cause, or to the end of October 2017. Survival analyses were performed using the Kaplan-Meier method and compared using the log-rank test for each group. For all statistical analyses, p<0.05 was considered statistically significant. The statistical package for social sciences (SPSS), version 23.0, software for Windows (IBM, Chicago, IL) was used for statistical analysis.

## Results

### Patient characteristics

The study consisted of 91 patients with cT1,2N0M0 OSCC treated with transoral resection of the primary tumor. Twelve patients also underwent elective neck dissection. The patients’ ages ranged from 33 to 88 years (median: 68 years). The clinicopathological characteristics of the cT1,2N0M0 OSCC patients are summarized in S1 Table. The median duration of follow-up was 90 months (range: 6–164 months).

### Mode of invasion, worst pattern of invasion, tumor budding, and regional metastasis

**Fig 4:**
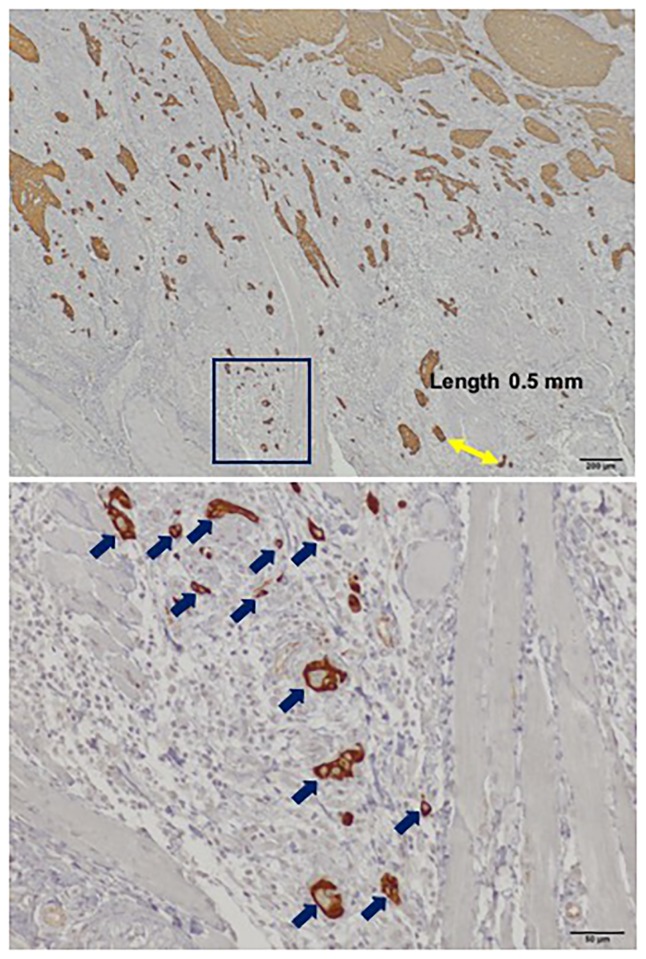
Photographs of immunohistochemical pancytokeratin staining show representative examples of grade 4D mode of invasion and worst pattern of invasion (WPOI)-4 at x40 magnification. The region in the rectangle is shown at x200 magnification in the lower panel, which shows 12 buds.

**Fig 5:**
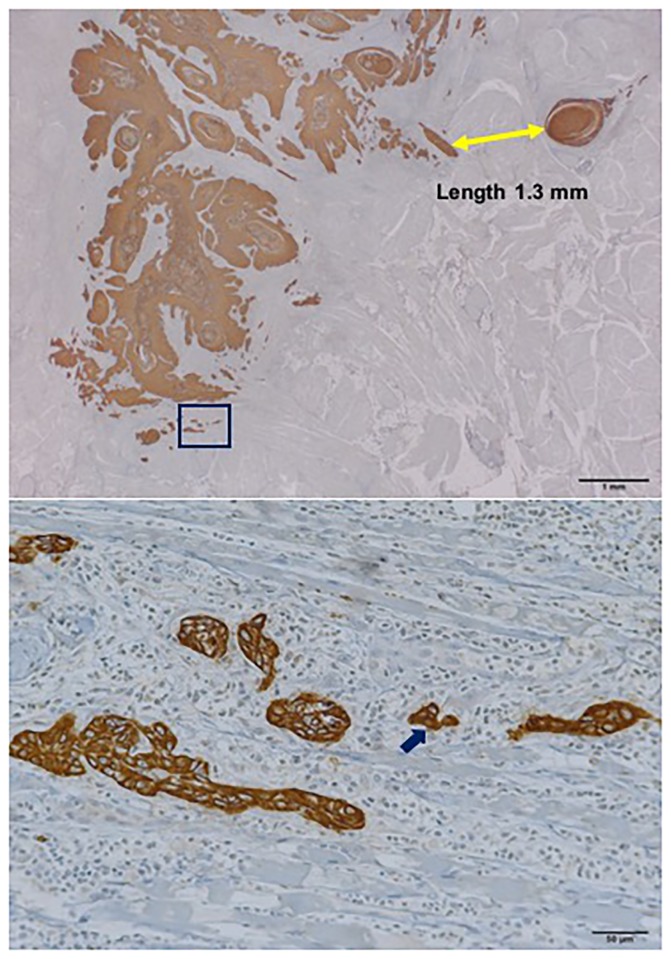
Photographs of immunohistochemical pancytokeratin staining show representative examples of grade 3 mode of invasion and WPOI-5 at x12.5 magnification. The yellow line measures a distance >1 mm between the main tumor and the next focus of dispersed islands. The region in the rectangle is shown at x200 magnification in the lower panel, which shows 1 bud.

In morphological assessment, a grade 1, 2, or 3 mode of invasion was present in 78 patients, grade 4C in 11 patients, and grade 4D in 2 patients. For WPOI, half of the patients (46/91, 50.5%) had small invasive tumor islands with a size <15 cells per island, including single-cell invasion (WPOI-4), 20 (22.0%) had invasive tumor islands larger than 15 cancer cells (WPOI-3), and 15 (16.5%) had tumor satellites at a distance of more than 1 mm from the main tumor (WPOI-5). Tumor buds were assessed easily with immunohistochemical sections as were the mode of invasion and WPOI (Figs 4 and 5). The median number of tumor buds was 2 (range: 0–18). Twenty-nine cases had more than 5 buds, of which 13 had more than 10 buds, and 16 had more than 5 buds but fewer than 10 buds at the deep invasive front of the tumor. Of the 62 low-intensity budding cases, no tumor bud was observed in 28.

Among the patients, 3 (3.2%) evidenced regional metastasis by elective neck dissection and 19 (20.8%) developed regional relapse without recurrence of the primary tumor from 3 to 34 months after transoral excision of the primary tumor. Correlations were tested among the clinicopathological parameters of the cT1,2N0M0 OSCC cases and regional metastasis. As expected, strong associations were observed for the mode of invasion (p<0.01) and intensity of tumor budding (p<0.01). Lymphovascular invasion was also significantly associated with regional metastasis (p = 0.04; S2 Table). Logistic regression analysis identified only tumor budding as an indicator of regional metastasis (hazard ratio (HR) = 3.05, 95% confidence interval (CI) = 0.29–5.30, p<0.01; S3 Table).

### Mode of invasion, worst pattern of invasion, tumor budding, and survival

**Fig 6:**
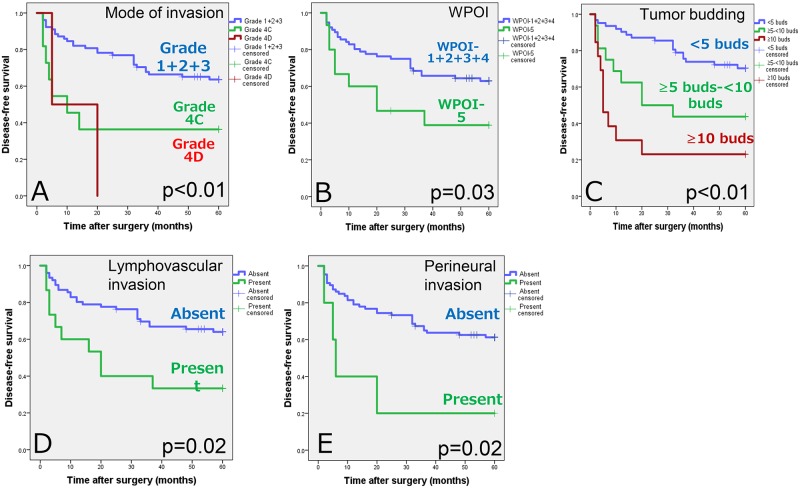
Kaplan-Meier survival curves by mode of invasion (A), worst pattern of invasion (WPOI) (B), tumor budding (C), lymphovascular invasion (D), and perineural invasion (E). 5-year disease-free survivals were 58.0% for grade 1+2+3 mode of invasion, 36.4% for grade 4C mode of invasion, 0.0% for grade 4D mode of invasion (A), 58.4% for WPOI-1+2+3+4, 33.3% for WPOI-5, 65.0% for low-intensity tumor budding (<5 buds), 37.5% for intermediate-intensity tumor budding (≥5 buds-<10 buds), 23.1% for high-intensity tumor budding (≥10 buds), 58.1% for absence of lymphovascular invasion, 33.3% for presence of lymphovascular invasion, 56.1% for absence of perineural invasion, and 20.0% for presence of perineural invsion in patients with clinically node-negative T1 and T2 oral squamous cell carcinoma.

The pathologic N stages were pN1 in 13 cases, pN2 in 7 cases, and pN2c in 2 cases. Eight patients had extracapsular spread (ECS) of metastatic tumors and 14 did not. One patient with ECS, the grade 4D mode of invasion, WPOI-5, and a high intensity of tumor budding developed regional relapse, and this patient had delayed therapeutic neck dissection. Among the 91 patients, 5 died of OSCC and 7 died of other causes, whereas 79 were alive at the end of the follow-up period. Of the 22 patients with regional metastases, 2 died of metastatic disease. The five-year disease-free survival rate was 54.2%. As illustrated in Fig 6, a striking difference in 5-year disease-free survival was observed among grade 1+2+3, grade 4C, and grade 4D modes of invasion (p<0.01), and between WPOI-1+2+3+4 and WPOI-5 (p = 0.03). The intensity of tumor budding was also a prognostic parameter for disease-free survival (p<0.01). With respect to the other histologic parameters, the presence and absence of lymphovascular invasion and perineural invasion showed equal statistical significance for disease-free survival (p = 0.01; S4 Table). A final stepwise model for Cox multivariate analysis identified the intensity of tumor budding at the invasive front of the tumor as the most highly significant and independent prognostic marker in patients with cT1,2N0M0 OSCC (HR = 2.18, 95% CI = 1.49–3.20, p<0.01; S5 Table).

### Interrelation between mode of invasion, worst pattern of invasion, and tumor budding

**Fig 7:**
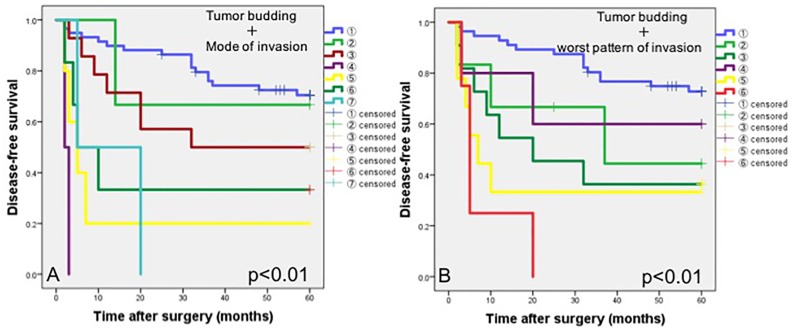
Kaplan-Meier curves of disease-free survival (DFS) in patients in combined groups with the intensity of tumor budding and mode of invasion (A) or worst pattern of invasion (WPOI) (B). In group A, the 5-year DFSs were 70.4% with low tumor budding and grade 1+2+3 mode of invasion ①, 66.7% with low tumor budding and grade 4C mode of invasion ②, 50.0% with intermediate tumor budding and grade 1+2+3 mode of invasion ③, 0% with intermediate tumor budding and grade 4C mode of invasion ④, 20.0% with high tumor budding and grade 1+2+3 mode of invasion ⑤, 33.3% with high tumor budding and grade 4C mode of invasion ⑥, and 0% with high tumor budding and grade 4D mode of invasion ⑦. In group B, the 5-year DFSs were 72.8% with low tumor budding and WPOI-1+2+3+4 ①, 44.4% with low tumor budding and WPOI-5 ②, 36.4% with intermediate tumor budding and WPOI-1+2+3+4 ③, 60.0% with intermediate tumor budding and WPOI-5 ④, 33.3% with high tumor budding and WPOI-1+2+3+4 ⑤, and 0% for high tumor budding and WPOI-5 ⑥.

The above-mentioned morphological assessments of the tumor are very similar; however, the mode of invasion is assessed based on the invasive morphology of the cancer cells or nests, not the cell number of the tumor island or nest. On the other hand, WPOI and tumor budding are assessed based on small tumor islands or nests. These assessments differ from the viewpoint of the cutoff value of cancer cells in the dispersed islands or nest and also the distance from the main tumor. Although patients with high intensities of tumor budding had poor survival, grouping done based on the intensity of tumor budding- combined with the mode of invasion or worst pattern of invasion, revealed that the 5-year survival rates of the patients with intermediate intensity of tumor budding plus the grade 4C mode of invasion, high intensity of tumor budding plus the grade 4D mode of invasion, and high intensity of tumor budding plus WPOI-5 had the poorest survivals (Fig 7). When the interrelations of these 3 assessments of invasive tumor patterns were examined, the relationship between the mode of invasion and WPOI, and the relationship between the mode of invasion and tumor budding showed statistically significant correlations, but the relationship between WPOI and tumor budding did not (p<0.01, p<0.01, and p = 0.14, respectively; S6 Table).

## Discussion

The current retrospective study showed four interesting results. First, it showed that higher intensities of tumor budding and grade 4C and grade 4D modes of invasion were correlated with regional metastasis in patients with cT1,2N0M0 OSCC. Second, it demonstrated that the intensity of tumor budding was an independent prognostic marker for disease-free survival in patients with cT1,2N0M0 OSCC. Third, it clearly identified that tumor budding was the most powerful biomarker among invasive tumor patterns, including the mode of invasion and worst pattern of invasion at the invasive tumor front of cT1,2N0M0 OSCC. Fourth, it is likely that immunohistochemical staining for pancytokeratin can be used by clinicians to correctly assess the invasive tumor pattern as well as the mode of invasion, WPOI, and tumor budding, resulting in therapeutic benefits for patients with cT1,2N0M0 OSCC. The two former findings of the current study are consistent with the results of the most recent up-to-date meta-analysis [5].

A large number of authors have reported that early stage node-negative OSCC developing regional metastasis has a poor prognosis [3, 13, 25]. There are two options for treatment of the neck in clinically node-negative OSCC. One is elective neck dissection and the other is wait-and-see. Some authors do not support the wait-and-see policy as patients who develop regional relapse during wait-and-see follow-up have greater morbidity after therapeutic neck dissection compared to elective neck dissection. The adverse outcome associated with delayed neck dissection can at least partly be explained by the fact that patients developing regional relapse have a more advanced nodal stage and higher incidence of ECS. Several studies have indicated that tumor budding might affect regional metastasis in patients with cT1,2N0M0 OSCC [26, 27, 28], Pedersen et al. [29] developed a semi-automated image-analysis algorithm, Digital Tumor Bud Count (DTBC), to evaluate tumor budding, and they demonstrated that their risk model was able to identify patients with regional metastasis in cT1,2N0M0 OSCC. Based on their results, the authors claimed that the use of their risk model would lead to fewer node-negative OSCC patients who receiving unnecessary neck dissection. Unfortunately, although we agree with them, laboratory procedures for investigation of tumor budding using digital image analysis are not commonly used at present. In our previous study that investigated regional metastasis-related predictors in 172 patients with T1-4N0 oral squamous cell carcinomas, grade 1 to 3 modes of invasion were shown a negative sign for predicted patients without regional metastasis, while grade 4C and 4D of modes of invasion were shown positive sign for predictive patients with regional metastasis [13]. Therefore, after these results are obtained, we pay careful attention to the follow-up strategy of watchful waiting, especially for patients having grades 4C and 4D modes of invasion with node-negative OSCC, who are followed up carefully at short intervals such as every two weeks with or without US, MR imaging, CT, and PET-CT in addition to careful physical examination for early detection and treatment of regional metastasis. As shown in the present study, we can achieve favorable survival for the grade 4C mode of invasion, and this may justify our follow-up policy and strategy of treatment as soon as possible when patients develop nodal relapse. Regarding to the number of distribution of mode of invasion, grade 1, 2, 3, 4C, and 4D in the previous study were 36 (21%), 47 (27%), 58 (34%), 17 (10%), and 14 (8%), respectively. Similarly, present study composed of grade 1: 10 (11%), grade 2: 30 (33%), grade 3: 38 (42%), grade 4C: 11 (12%), and grade 4D: 2 (2%). Grade 1 to 3 mode of invasion were composed of 82% in the previous study and 86% in the present study. Although grade 4D mode of invasion was somewhat small number of cases (2%) in the present study compared to 8% in the previous study, the case distribution in the previous and present study was very similar.

A meta-analysis evidenced a significant correlation between the presence of more than 5 buds and poor disease-free survival (HR = 1.83, 95%CI = 1.34–2.50) or poor overall survival (HR = 1.88, 95%CI = 1.25–2.83) in OSCC [5]. Tumor budding has been reported to be a novel prognostic marker for the early stage of malignancies such as esophageal cancer [30], endometrial cancer [31], and tongue cancer [32]. Xie et al. [27] demonstrated that tumors with more than 5 buds were associated with microscopic tumor involvement in the regional lymph nodes and poor overall survival in early stage tongue squamous cell carcinoma. However, the estimated 95% CI ranged from 1.23 to 25.38. On the other hand, our representative disease-free survival was calculated based on three categories: low intensity (<5 buds/field), intermediate intensity (≥5~<10 buds/field) and high intensity (≥10 buds/field) according to the ITBCC 2016 recommendations [24]. This assessment using these 2 cutoff points will be valuable for clinical use or application because the results are supported by strong statistical evidence.

Although several different studies have reported the relationships among regional metastasis in patients with node-negative OSCC, the mode of invasion [13,33], and tumor budding [34], little is known about the clinical and prognostic value of the combination of the mode of invasion, WPOI, and tumor budding at the invasive tumor front. The present study revealed that the mode of invasion was well correlated with WPOI and tumor budding. However, WPOI was not correlated with tumor budding. The mode of invasion and WPOI are assessed by invasive morphologic patterns based on pushing/expanding invasion or infiltrating invasion by single or small clusters of tumor cells, with diffuse or cord-like invasive patterns observed depending on the mode of invasion. In WPOI assessment, infiltrating tumor nests are divided into two groups, large tumor islands (>15 cells) and small tumor islands (≤15 cells). In addition, WPOI assessment is characterized by measurement of the distance from the main tumor to the tumor island. This criterion of assessment is not required for the mode of invasion or tumor budding. In addition, tumor budding is assessed differently from WPOI, on the basis of only the numbers of single cells or tumor islands of less than 5 tumor cells. Therefore, the difference between WPOI and tumor budding for regional metastasis in clinically node-negative OSCC may be due to their different criteria.

Most studies have evaluated tumor budding using H&E staining. Indeed, H&E staining has been widely accepted as a histopathologic diagnostic tool for diseases in the field of surgical pathology during the past century. However, accurate diagnostic observation of the microscopic findings requires long training and experience. Therefore, we used immunohistochemistry for the evaluation of budding foci and counting in this study. Leão et al. [35] suggested that assessment of tumor budding should be performed by pan-cytokeratin immunostaining, considering its higher reproducibility, greater replicability, and lower difficulty compared to H&E staining. We also easily observed the mode of invasion and WPOI in the present study. We believe that evaluation of tumor budding by immunohistochemistry with an anti-pancytokeratin monoclonal antibody can shorten the working time and provide better reproducibility of results than with H&E staining, especially for surgeons.

In conclusion, the intensity of tumor budding may be a novel diagnostic and prognostic biomarker, as well as a therapeutic tool, for patients with cT1,2N0M0 OSCC. Our results show that tumor budding with more than five buds and a grade 4C or 4D mode of invasion is observed more frequently in patients with cT1,2N0M0 OSCC with regional metastasis, through only a high intensity of tumor budding showed a significant correlation with regional metastasis in multivariate analysis. Hence, the authors would like to propose the following diagnostic and treatment strategies for identifying regional metastasis in patients with cT1,2N0M0 OSCC: (1) assess tumor budding, the mode of invasion in pancytokeratin-immunostained biopsied and surgically resected tissue sections, (2) identify budding with more than five buds and grade 4C and 4D modes of invasion and, if these features are present, (3) conduct careful wait-and-see follow-up at short intervals such as every week or every two weeks for 6 months after primary ablation surgery with or without the use of imaging modalities, (4) if there are symptoms of developing nodal relapse, perform neck dissection as soon as possible, but (5) if budding with more than ten buds and the grade 4D mode of invasion, or WPOI-5 are observed in the same section, we recommend wider resection of the primary tumor with elective neck dissection.

## Supporting information

S1 Table Patient and tumor characteristics: T1,2N0 oral squamous cell carcinoma. (XLSX)

S2 Table Associations between clinical and histopathologic variables and regional lymph node metastasis. (XLSX)

S3 Table Logistic regression analysis to assess associations between tumor invasive patterns and regional lymph node metastasis in cT1,2N0M0 oral squamous cell carcinoma. (XLSX)

S4 Table Univariate analyses by patient and tumor characteristics for 5-year disease-free survival. (XLSX)

S5 Table Stepwise proportional hazards regression. (XLSX)

S6 Table Interrelationships among mode of invasion, worst pattern of invasion, and tumor budding. (XLSX)
